# Potentially Preventable Mortality in the Pediatric Intensive Care Unit: Findings from a Retrospective Mortality Analysis

**DOI:** 10.7759/cureus.7358

**Published:** 2020-03-21

**Authors:** Qalab Abbas, Fozia Memon, Parveen Laghari, Ali Saleem, Anwar Haque

**Affiliations:** 1 Pediatrics and Child Health, Aga Khan University Hospital, Karachi, PAK; 2 Pediatrics, Aga Khan University Hospital, Karachi, PAK; 3 Pediatrics, The Indus Hospital, Karachi, PAK

**Keywords:** mortality, analysis, retrospective, pediatric intensive care unit

## Abstract

Objective

The goal of this study was to estimate the proportion and causes of potentially preventable mortality among critically ill children admitted to the pediatric intensive care unit (PICU).

Methods

The medical records of all patients who died in the PICU (age range: one month to 16 years) between January 2014 and December 2015 were evaluated by two independent reviewers to determine whether there had been any delayed recognition of deteriorating conditions, delayed interventions, unintentional/unanticipated harm, medication errors, adverse reactions to transfusions, and hospital-acquired infections that could have resulted in unanticipated death. Preventability was labeled on a 6-point scale.

Results

During the study period, 92 of 690 patients did not survive [median age: 60 months, interquartile range (IQR): 114]. The median Pediatric Risk of Mortality (PRISM) III score was 17 (IQR: 6). Major diagnostic categories included sepsis (n = 29, 35%), central nervous system diseases (n = 16, 17%), oncological/hematological diseases (n = 6, 6%), cardiac diseases (n = 4, 4%), and miscellaneous conditions. None of the deaths had definitive or strong evidence of preventability. Four (4.3%) patients were in category 4 (i.e., possibly preventable, >50/50 chance), 15 (16.3%) in category 3 (possibly preventable, <50/50 chance), 28 (30.4%) had some evidence of preventability, and 45 (49.0%) were labeled as definitely not preventable. Late identification (diagnostic error) of the worsening condition in four (21.0%) patients, slow intervention in six (31.6.0%), and hospital-acquired infections in 10 (52.6%) were found to be related to potentially preventable mortality.

Conclusions

Preventable diagnostic errors and nosocomial infections (NIs) are major contributors to preventable mortality. Structured mortality analysis provides actionable information for future preventive strategies. Improvement in care processes, including clinical decision support systems, could help reduce preventable mortality rates.

## Introduction

The pediatric intensive care unit (PICU) is a fast-paced, stressful environment prone to regular occurrence of errors, which can lead to suboptimal patient outcomes [1,2]. According to Kohn et al., 98,000 people die each year in US hospitals due to preventable medical errors [3]. These medical errors were categorized as diagnostic, treatment-related, and others, with most of them being attributed to faulty systems, processes, or conditions [3]. Likewise, after that report, data from several other PICUs showed that up to 60% of patients admitted to the PICU experience some form of an adverse event during their stay [2,4-6]. These studies have highlighted the problems of diagnostic errors, delayed interventions, and technical problems with procedures as major forms of preventable harms [4,7]. PICUs in Pakistan are a scarce resource, as evidenced by a significant unmet need [8]. Lack of trained faculty, staff, and proper equipment and systems may cause high mortality and greater complication rates compared to other developing and developed countries, impacting patient outcomes [1]. In a country with a high mortality rate for patients under the age of five years, developing the quality of care is a priority. Improved awareness of and information on patient safety factors will facilitate the planning of interventions that can reduce related preventable harms.

## Materials and methods

This study was conducted at the PICU of Aga Khan University Hospital (AKUH), a tertiary care hospital in Karachi, Pakistan. The PICU at AKUH is a four-bed closed multidisciplinary unit with 350 admissions annually. It is staffed by a senior resident and a fellow 24 hours a day, supervised by three trained pediatric intensivists who are similarly fully available to provide patient care. The patient-to-nurse ratio is 1:1, and all physicians and nurses are Pediatric Advanced Life Support (PALS)-certified. The PICU admits patients with a wide variety of diagnoses including complex medical conditions as well as postoperative surgical and trauma patients. The PICU has all the facilities available except extracorporeal membranous oxygenation. Patients are admitted from the emergency department (ED) directly after presentation or after being referred to our center from nearby or remote regional hospitals with occasional travel time as long as 12 hours.

The PICU of AKUH follows standard management guidelines for different diseases, such as PALS guidelines for shock management and Pediatric Acute Lung Injury Consensus Conference (PALICC) guidelines for pediatric acute respiratory distress management, among others [9-11].

We conducted a retrospective chart review of all children (age range: one month to 16 years) who died in the PICU between January 2014 and December 2015 after approval from the hospital’s ethical review committee. Patients were identified through the hospital’s health information management system using International Statistical Classification of Diseases and Related Health Problems, 10th revision (ICD-10) coding for mortality, and were confirmed by cross-referencing PICU logbooks and the hospital’s mortality meeting records.

Data was collected through study questionnaires using inpatient medical records by two independent reviewers (QA and FM) with over one year of experience working in the PICU. They were trained, qualified pediatricians despite not being primarily responsible for direct patient care during the study period. Following the approach of Dijkema et al., we labeled preventability as “an event [mortality in our study] that would not have occurred if the patient had received ordinary standards of care appropriate for the time of study” [12]. Standard of care was defined as accepted patient care by an average practitioner using currently available evidence. The reviewers underwent half-a-day of training for the data collection procedure and were asked to carefully review the detailed medical charts of patients to determine whether there had been any problem in care that had contributed to the patient’s expected/unexpected mortality. Patients’ entire medical records were reviewed including physician notes, nursing, and allied health professionals’ notes, critical care flow sheets (used for documenting vital signs, ventilatory parameters, intake/output, and hemodynamic data), drug prescription and administration charts, blood transfusion charts, operative reports where applicable including all invasive procedure notes, as well as laboratory and radiological test results. Whenever a problem in care was found, the problem’s nature, timing, and associated causative/contributory factors were determined. The decision of labeling a death as preventable was a two-stage process. First, the reviewers were asked to assess the care process and judge whether there had been any problem that had contributed to the patient’s death. Harm was defined as an act of commission or omission or unintended complications of healthcare. It was divided into delayed recognition of a patient’s problem (diagnostic errors, as defined by Cifra et al. [7]), slow intervention, unintended/unexpected harm to the patient during a procedure (inappropriate technical procedure), and medication error (i.e., an error occurring in the process of medication delivery including medication prescription, administration, or monitoring). Findings were then matched for consistency, and only findings of mutual agreement were considered. In the second stage, for each case where reviewers found a problem in care leading to a patient’s death, the preventability of death was assessed based on a previously validated 6-point scale [13] (Table 1).

**Table 1:** Scale of preventability

Description of scale of preventability used	Point	Category
Virtually no evidence of preventability	1	1
Slight evidence of preventability	2	2
Preventability quite likely (<50/50)	3	3
Preventability quite likely (>50/50)	4	4
Strong Evidence for preventability	5	5
Virtually strong evidence of preventability	6	6

Mortality was labeled as preventable when the score was >3. Both reviewers scored each mortality separately, and a final decision was confirmed in cases where both reviewers had similar findings. In the event of a disagreement, a third reviewer (AH) reviewed the file and provided a final assessment, which was recognized as the final decision. Standard definition was used for the diagnosis of nosocomial infections (NIs), blood transfusion reactions, and multiple organ dysfunction syndromes (MODS) [14-16]. Demographic details, other relevant clinical details (e.g., source of admission, the number of organ involvement at presentation, MODS, NIs, drug reaction, transfusion reaction, an episode of cardiopulmonary resuscitation, mode of death) were recorded. Pertinent laboratory data were also collected and incorporated into the main analysis wherever needed [i.e., defining MODS and Pediatric Risk of Mortality (PRISM)]. Admission diagnostic categorization was done based on the primary or predominant system involved. Data were analyzed using IBM SPSS Statistics for Windows, Version 20.0 (IBM Corp., Armonk, NY). Results are presented as mean and standard deviation for continuous variables and frequency and percentage for categorical variables.

## Results

During the study period, a total of 92 of 690 patients died with an overall mortality rate of 133 per 1,000 PICU admissions. The study population had a median age of 60 months [interquartile range (IQR): 114]; 52 (56.0%) were males, and the median PRISM III score was 17 (IQR: 6).

Major diagnostic categories at admission included sepsis (n = 29, 31.5%), central nervous system diseases (n = 16, 17.4%), oncological/hematological diseases (n = 6, 6.5%), cardiac diseases (n = 4, 4.3%), and miscellaneous conditions. Admission source included the ED (n = 72, 78.2%), pediatric ward, step-down unit (n = 19, 20.7%), and operating room (one patient). MODS at admission was present in 88 patients (95.6%). Among them, 16 (17.4%) children died within 24 hours from PICU admission (Table 2).

**Table 2:** Clinico-demographic features of patients admitted to the pediatric intensive care unit during the study period (n = 690) IQR: interquartile range; MODS: multiple organ dysfunction syndrome; CPR: cardiopulmonary resuscitation; PICU: pediatric intensive care unit; PRISM III: Pediatric Risk of Mortality III score

Variables			Survived (n = 598)	Expired (n = 92)
Age in months, median (IQR)			65 (100)	60 (114)
Gender		Male, n (%)	358 (60)	52 (56)
		Female, n (%)	240 (60)	40 (44)
PRISM III score, median (IQR)			8 (7)	17 (6)
Disease diagnosis categorization at admission, n (%)	Central nervous system		120 (20)	16 (17.3)
	Cardiovascular system		132 (22)	4 (4.3)
	Respiratory system		90 (15)	5 (5.4)
	Hematology/oncology		47 (08)	6 (6.5)
	Infection/sepsis/MODS		84 (14)	29 (31.5)
	Gastrointestinal/hepatic		41 (7)	18 (19.5)
	Miscellaneous		84 (14)	14 (15.2)
CPR during PICU stay, n (%)			9 (1.5)	16 (17.3)
Source of admission, n (%)		Emergency department	478 (80)	72 (78.2)
		Special care unit	61 (10)	14 (15.2)
		Ward	24 (4)	5 (5.4)
		Operating room	35 (6)	1 (1)
MODS at admission, n (%)			30 (5)	85 (92)
Length of stay, median (IQR)			3.2 (3.5)	3 (4)
Any growth in bacterial culture prior to transfer during PICU stay, n (%)			18 (3)	42 (45.6)
Died within 24 hours, n (%)			0	16 (17.4)

Four (4.3%) patients were in category 4 (i.e., possibly preventable, >50/50 chance), 15 (16.3%) in category 3 (possibly preventable, <50/50 chance), 28 (30.4%) had some evidence of preventability, and 45 (49.0%) were labeled as definitely not preventable (Table 3).

**Table 3:** Scale of preventability in study population (n = 92) *Category 3; **Category 4

Description of scale of preventability used	Frequency (%)
Virtually no evidence of preventability	45 (49)
Slight evidence of preventability	28 (30.4)
Preventability quite likely (<50/50)*	15 (16.3)
Preventability quite likely (>50/50)**	4 (4.3)
Strong evidence of preventability	0
Virtually strong evidence of preventability	0

In category 4, one child had acute respiratory distress syndrome after community-acquired pneumonia; delayed provision of adequate respiratory support was deemed to be the likely cause of worsening hypoxia leading to death. One patient had a ventricular septal defect presenting with congestive cardiac failure. Delayed recognition of the worsening condition and central line-associated bloodstream infection (CLABSI) led to death. One patient had dengue hemorrhagic shock. Delayed resuscitation (i.e., delayed intervention) was found to be the cause of death. One patient presented with severe sepsis. Despite the initial improvement, the patient eventually died due to CLABSI.

In category 3, eight children deteriorated after the development of a Gram-negative CLABSI (four had carbapenem-resistant Enterobacteriaceae, another two had multidrug-resistant Acinetobacter and Enterococcus). Of those, one patient also developed transfusion-associated acute lung injury, and one developed methotrexate toxicity after receiving high-dose methotrexate for osteosarcoma. The delayed intervention was thought to be the reason for mortality in three patients and delayed recognition in four others. Eight of these 15 children were shifted from ward/high dependency units, while seven were admitted from the ED.

In category 2, 17 patients had MODS at presentation with evidence of delayed presentation/transport, and 10 patients had prolonged ED stays due to the unavailability of PICU beds. Five patients were noncompliant with treatment for their disease (e.g., tuberculosis or diabetes; Table 4).

**Table 4:** Details of patients judged as having preventable mortality Category 3: preventability quite likely (<50/50); category 4: preventability quite likely (>50/50)

Serial No	Primary diagnosis	Reason for being judged preventable	Preventability scale (category)
1	Acute respiratory distress syndrome	Delayed intervention	4
2	Autoimmune hepatitis	Delayed intervention	3
3	Congestive cardiac failure	Delayed recognition	4
4	Dengue hemorrhagic shock	Delayed intervention	4
5	Disseminated tuberculosis	Delayed recognition	3
6	Acute liver failure	Inappropriate procedure (hospital-acquired infection)	3
7	Fulminant myocarditis	Inappropriate procedure (hospital-acquired infection)	3
8	Hemophagocytic lymphohistiocytosis	Inappropriate procedure (hospital-acquired infection)	3
9	Osteosarcoma, methotrexate toxicity	Drug error, delayed recognition	3
10	Aplastic anemia	Inappropriate procedure (hospital-acquired infection), delayed recognition	3
11	Red cell aplasia	Delayed intervention	3
12	Acute respiratory failure	Delayed intervention	3
13	Severe sepsis	Inappropriate procedure (hospital-acquired infection)	4
14	Subarachnoid hemorrhage	Inappropriate procedure	3
15	Down syndrome with pulmonary hypertensive crises	Delayed recognition	3
16	Acute respiratory distress syndrome	Inappropriate procedure (hospital-acquired infection)	3
17	Septic shock	Inappropriate procedure (hospital-acquired infection)	3
18	Diabetic ketoacidosis with acute kidney injury	Delayed recognition, inappropriate procedure (hospital-acquired infection)	3
19	Severe pneumonia with acute respiratory failure	Inappropriate procedure (hospital-acquired infection)	3

## Discussion

In this two-year mortality analysis, 20% of mortality was deemed potentially preventable. This rate is lower than previously reported in the region and globally [7,17-20]. NIs, delayed recognition (i.e., diagnostic errors), and delayed intervention were common occurrences in our review. Compared to the findings by Cifra et al., the rate of diagnostic errors in our patient cohort was high (n = 4, 21%) [7]. The other reason was NI (10/19, 52.6%), which was in line with the results of a study done in a South African PICU [21]. While delayed intervention was found to be the cause of mortality in six of 19 patients (31.6%), similar to the results of other reports, NIs are potentially preventable; however, because of their complexity, it is difficult to label a specific error as a causative factor [6,7,19]. Some of these patients were admitted from the ward, which emphasizes the need and importance of early recognition and timely intervention on a deteriorating condition, and the important role of rapid response teams (RRTs). We have a very active RRT available 24 hours a day, but factors such as hospital system barriers and care team perceptions can have an impact on their roles [22,23]. We have previously shown a reduction in on-floor mortality associated with RRT. We believe additional strengthening with ongoing training can help in this regard [22]. Another important aspect is infection prevention and control (IPC). Though we do have standard practices and protocols for IPC and have shown an associated reduction in NI, ongoing training remains essential [24]. We have developed a one-day IPC workshop to train PICU nurses on standard IPC practices, which will help reduce potentially preventable mortality. The interrelation of illness severity, procedure exposure, and mortality is complex. This presented great difficulty for the reviewers in our study, and they found it challenging to differentiate between preventable and uncontrollable situations. This judgment also varies with the adequacy of medical records and reviewer training and experience.

Our study is the first comprehensive report of the preventability of PICU mortality originating from Pakistan. The study is limited by its design (i.e., retrospective, single-center report). Furthermore, autopsies are rarely performed at our institution, which prevents access to significant information on patients’ disease processes. Additionally, the quality of care before PICU admission, a factor that can affect patient outcomes, was beyond our control.

## Conclusions

Preventable diagnostic errors and NIs are major contributors to preventable mortality. Structured mortality analysis provides actionable information for future preventive strategies. Improvement in care processes, including clinical decision support systems, could help reduce preventable mortality rates. We should strive to learn from every single mortality to improve the quality of care.
